# A Comprehensive Safety Assessment of *Ralstonia eutropha* H16 for Food Applications: Integrating Genomic, Phenotypic, and Toxicological Analyzes

**DOI:** 10.3390/microorganisms13061323

**Published:** 2025-06-06

**Authors:** Xiaoyan You, Shuxia Song, Bing Li, Hui Wang, Le Zhang, Xiangyang Li, Junliang Chen, Zhiguang Zhu, Guoping Zhao

**Affiliations:** 1 Henan Engineering Research Center of Food Microbiology, College of Food and Bioengineering, Henan University of Science and Technology, Luoyang 471023, China; asongshuxia@163.com (S.S.); zhangle@tib.cas.cn (L.Z.); xiangyang_224@163.com (X.L.); junliangchen@126.com (J.C.); 2 National Center of Technology Innovation for Synthetic Biology, Tianjin Institute of Industrial Biotechnology, Chinese Academy of Sciences (CAS), Tianjin 300308, China; libing@tib.cas.cn (B.L.); wanghuih@tju.edu.cn (H.W.); zhu_zg@tib.cas.cn (Z.Z.); 3 Haihe Laboratory of Synthetic Biology, Tianjin 300308, China; 4 School of Life Sciences, Faculty of Medicine, Tianjin Key Laboratory of Function and Application of Biological Macromolecular Structures, Tianjin University, Tianjin 300072, China; 5 CAS-Key Laboratory of Synthetic Biology, CAS Center for Excellence in Molecular Plant Sciences, Institute of Plant Physiology and Ecology, Chinese Academy of Sciences, Shanghai 200032, China; 6 CAS Key Laboratory of Quantitative Engineering Biology, Shenzhen Institute of Synthetic Biology, Shenzhen Institute of Advanced Technology, Chinese Academy of Sciences, Shenzhen 518055, China; 7 Engineering Laboratory for Nutrition, Shanghai Institute of Nutrition and Health, Chinese Academy of Sciences, Shanghai 200031, China

**Keywords:** *Ralstonia eutropha* H16, safety assessment, phenotypic analysis, acute and subacute toxicity

## Abstract

*Ralstonia eutropha* H16, a metabolically versatile bacterium, has gained prominence as a microbial platform for sustainable bioproduction. While its capabilities in synthesizing single-cell proteins and biodegradable materials are well documented, comprehensive strain-level safety evaluations remain insufficient for food-grade applications. This study systematically assessed the safety of *R. eutropha* H16 through genomic, phenotypic, and toxicological analyzes. Genomic analyzes revealed the absence or minimal presence of virulence factors and antibiotic resistance genes, aligning with microbiological safety standards. Phenotypic investigations demonstrated a limited gastric fluid tolerance (pH 2.5, survival rate 25.70% after 3 h) and intestinal fluid persistence (pH 8, 44.67% viability after 3 h), coupled with an exceptional bile salt tolerance (0.2% *w*/*v*). Antioxidant assays confirmed the fermentation broth specifically scavenges DPPH free radicals (14.60 ± 1.24 μg Trolox/mL), whereas bacterial suspensions and cell-free supernatants exhibited a strong hydroxyl radical scavenging (>90 U/mL) and superoxide anion inhibition (>100 U/L). Acute toxicity testing indicated no mortality or histopathological abnormalities, with an LD_50_ value exceeding 1 × 10¹¹ CFU/kg. Subacute toxicity studies (28-day, 1 × 10^8^–1 × 10^10^ CFU/kg) revealed no significant effects on growth, hematology, or organ function. Minor alterations in serum biochemistry might be attributed to physiological adaptation. Subacute exposure induced transient serum ALT fluctuations without hepatorenal dysfunction, while maintaining hematological parameters within physiological ranges. Collectively, these results substantiate the safety of *R. eutropha* H16 for food-related applications while underscoring the necessity of strain-specific risk assessments for industrial microbial platforms.

## 1. Introduction

As the global population is projected to reach 9.7 billion by 2050, coupled with the escalating impacts of climate change, the need for innovative and sustainable solutions in food production has never been more urgent [1]. Among the promising alternatives gaining attention is microbial biotechnology, which offers a transformative approach to addressing food security and environmental sustainability. At the forefront of this revolution is *Ralstonia eutropha* H16, also known as *Cupriavidus necator* H16, a Gram-negative bacterium belonging to the β-subclass of *Proteobacteria* [2]. This microorganism stands out for its extraordinary metabolic versatility, capable of switching between autotrophic and heterotrophic growth modes depending on environmental conditions [3]. Such adaptability has positioned *R*. *eutropha* H16 as a versatile industrial platform, comparable to well-established workhorses like *Escherichia coli* and *Saccharomyces cerevisiae* [4].

One of the most compelling applications of *R*. *eutropha* H16 lies in the production of single-cell proteins (SCPs), which are increasingly recognized as a sustainable alternative to conventional animal-derived proteins [5]. SCPs derived from *R*. *eutropha* H16 offer several distinct advantages. Firstly, they exhibit a high digestibility, with studies showing an up to 93% digestibility in animal models—comparable to casein, a gold standard in protein quality [6]. Secondly, *R*. *eutropha* H16 boasts a superior production efficiency, characterized by a higher protein content, shorter production cycles, and minimal operational constraints, making it a key player in addressing global protein supply imbalances [7]. Thirdly, this bacterium contributes to environmental sustainability by efficiently recycling organic waste into high-quality protein biomass that rivals fishmeal and pork in nutritional value, while simultaneously reducing carbon dioxide emissions [8]. These attributes make *R*. *eutropha* H16 not only a solution for food security but also a powerful tool for waste-to-resource conversion.

Beyond SCP production, *R*. *eutropha* H16 has demonstrated remarkable potential in food processing and biotechnological industries. Through genetic engineering, this bacterium can synthesize essential amino acids such as L-isoleucine and L-valine, which enhance the nutritional and sensory qualities of food products [9]. It also serves as an effective biocatalyst, facilitating critical food processing reactions like starch hydrolysis and proteolysis, thereby improving efficiency and product quality [10]. Furthermore, *R*. *eutropha* H16 can ferment industrial byproducts, such as molasses, into polyhydroxybutyrate (PHB), a biodegradable plastic ideal for eco-friendly food packaging [11]. By repurposing waste streams, PHB contributes to pollution reduction and aligns with global efforts toward a circular economy [12]. Collectively, these diverse applications highlight the immense potential of *R*. *eutropha* H16 to advance both food production systems and sustainable industrial practices.

Despite its growing prominence, a significant knowledge gap remains regarding the safety of *R*. *eutropha* H16 at the strain level. While research has predominantly focused on its metabolic engineering capabilities, toxicological characterization has received limited attention. In 2019, the European Food Safety Authority (EFSA) added *Cupriavidus necator* to its Qualified Presumption of Safety (QPS) list, but restricted its use to production purposes only, assuming rigorous heat treatment and purification during manufacturing [13]. However, microbiologically derived food products require thorough safety assessments to mitigate potential risks and ensure compliance with industrial biosafety standards [14]. The variability within microbial species further complicates safety evaluations; for example, while some strains of *E*. *coli* are highly pathogenic, others are harmless or even beneficial [15]. This underscores the necessity of refining safety assessments to the strain level to ensure the reliable and safe deployment of microbial platforms.

To address this critical need, our study undertakes a comprehensive evaluation of the safety profile of *R*. *eutropha* H16 through a multi-faceted approach. We conducted genomic analyzes to identify potential virulence factors and antibiotic resistance genes, phenotypic assessments to evaluate antibiotic sensitivity and gastrointestinal tolerance, and acute and subacute toxicity tests to investigate biological safety. By integrating these methodologies, we aim to establish a robust framework for validating the suitability of *R*. *eutropha* H16 as a safe microbial platform for food-related applications. This research not only fills a significant gap in the literature but also highlights the importance of strain-specific risk assessments in ensuring the safe adoption of industrial microorganisms. Ultimately, our findings contribute to advancing sustainable and secure food production systems, paving the way for a more resilient future in the face of global challenges.

## 2. Materials and Methods

### 2.1. Genomic Properties of R. eutropha H16

The genome sequence data of *R*. *eutropha* H16 (Genbank accession number: GCA_004798725.1) were subjected to multidimensional bioinformatics analysis. Sequence homology searches were performed using the Diamond program (version 2.1.9), with optimal matches identified based on alignment parameters. Functional annotation was systematically conducted through integration with the Kyoto Encyclopedia of Genes and Genomes (KEGG), Cluster of Orthologous Groups (COG), and Gene Ontology (GO) databases. Carbohydrate metabolism-associated enzymes were predicted using the Carbohydrate-Active Enzymes Database (CAZy). Biosafety evaluation employed a tripartite validation framework: virulence potential was assessed via screening against the Virulence Factor Database (VFDB), Microbial Virulence Factor Database (MvirDB), and Pathogen–Host Interaction Database (PHI-base); antibiotic resistance determinants were analyzed using the Comprehensive Antibiotic Resistance Database (CARD), Antibiotic Resistance Gene Database (ARDB), and Resistance Gene Family Database (Resfam). All database comparisons adhered to stringent criteria: sequence identity ≥ 80%, length coverage ≥ 70%, and e-value < 10^−5^.

### 2.2. The Physiological Features Analysis of R. eutropha H16

#### 2.2.1. Bacterial Cultivation Conditions

*R*. *eutropha* H16 was cultivated in Lysogeny broth (LB) supplemented with gentamicin sulfate (10 μg/mL final concentration). Cultures were incubated at 30 °C with orbital shaking (200 rpm) for 16–20 h to achieve the exponential growth phase (OD_600_ ≈ 0.8). Prior to experimentation, single colonies were isolated on LB agar plates and subcultured twice in fresh medium to ensure physiological consistency.

#### 2.2.2. Antibiotic Susceptibility Testing

Susceptibility to 10 antibiotics (gentamicin, ampicillin, chloramphenicol, streptomycin, clindamycin, tetracycline, kanamycin, ciprofloxacin, vancomycin, and erythromycin) was determined via disc diffusion assay following Clinical and Laboratory Standards Institute (CLSI) guidelines. Briefly, bacterial suspensions (1 × 10^8^ CFU/mL) were spread on LB agar, and antibiotic-impregnated discs (Bkmamlab, Changsha, China) were applied. After incubation at 30 °C for 12 h, inhibition zones were measured and classified as resistant (R), susceptible (S), or intermediate (I) based on CLSI breakpoints. The criteria for the determination of drug sensitivity are shown in Supplementary Table S1 [16].

#### 2.2.3. Acid and Bile Salt Tolerance

For acid tolerance, cultures (2% *v*/*v* inoculum) were incubated in LB medium adjusted to pH 2.0, 3.0, or 4.0 using HCl/NaOH. Bile salt tolerance was assessed in LB supplemented with 0.1–0.3% (*w*/*v*) bile salts (Mreda, Beijing, China, Cat. #M048527). Growth kinetics were monitored at 30 °C for 24 h using an automated growth curve analyzer (High-density Growth Curve Detector, Jieling Instrument Manufacturing (Tianjin) Co., Ltd., Tianjin, China) [17].

#### 2.2.4. Simulated Digestive Fluid Survival

Simulated gastric fluid (SGF) comprised 0.3% pepsin (Biotopped, Beijing, China, Cat. #P6390C) and 0.5% NaCl (pH 2.5), while simulated intestinal fluid (SIF) contained 0.1% trypsin (Sigma-Aldrich, Shanghai, China, Cat. #8049-47-6), 0.3% bile salts, and 0.5% NaCl (pH 8.0). *R*. *eutropha* H16 suspensions (1 × 10^8^ CFU/mL) were incubated in SGF/SIF at 37 °C, with viable counts determined by plate enumeration at 0 and 3 h post-exposure [18].

#### 2.2.5. Antioxidant Activity Assays

Cells were harvested by centrifugation (4000× *g*, 10 min, 4 °C), and supernatants were filter-sterilized (0.22 μm) to obtain cell-free extracts. Pellets were resuspended in phosphate-buffered saline (PBS; OD_600_ = 1.0). Cell lysates were prepared via sonication (150 W, 5 s pulse/10 s pause, 20 min total) followed by centrifugation (12,000× *g*, 10 min) [19]. Antioxidant capacities (DPPH, hydroxyl radical, and superoxide anion scavenging) of cell suspensions, supernatants, and lysates were evaluated using commercial kits (Nanjing Jiancheng Bioengineering Institute, Nanjing, China).

#### 2.2.6. Biofilm Formation Quantification

Biofilm formation was assessed in 96-well polystyrene plates inoculated with *R*. *eutropha* H16 (1 × 10^6^ CFU/mL in LB) and incubated at 37 °C for 24 h. Non-adherent cells were removed by phosphate-buffered saline (PBS) washing, and adherent biofilms were fixed with methanol and stained with 1% crystal violet. Absorbance (OD₅₉₅) was measured using a microplate reader (Infinite M Plex, Tecan Trading Co., Ltd., Shanghai, China). Biofilm strength was categorized as non-(OD ≤ OD_C_), weak (OD_C_ < OD ≤ 2OD_C_), moderate (2OD_C_ < OD ≤ 4OD_C_), or strong (OD > 4OD_C_), where OD_C_ = mean negative control OD + 3 × standard deviation [20].

#### 2.2.7. Self-Aggregation and Hydrophobicity Evaluation

Cells were pelleted (4000× *g*, 10 min, 4 °C), washed twice in PBS, and resuspended to OD_600_ = 1.0 (~1 × 10^9^ CFU/mL). For self-aggregation, suspensions were incubated statically, and absorbance (OD_600_) was recorded at 0–24 h [21]. Hydrophobicity was evaluated by mixing bacterial suspensions with equal volumes of xylene, hexadecane, or octane, followed by an absorbance measurement of the aqueous phase after 1–8 h of incubation. Carefully aspirate the lower aqueous phase and measure absorbance [22]. Self-aggregation/hydrophobicity was calculated as follows:

$$Self-aggregation/Hydrophobicity\%=1-\frac{P0-Pt}{P0}\times 100\%$$

where *P*0 and *Pt* denote absorbance at initial and experimental timepoints, respectively.

### 2.3. Animals’ Experiment

#### 2.3.1. Animal Husbandry

Six to eight-week-old Sprague Dawley rats (180–220 g; Beijing Viton Lihua Laboratory Animal Technology Co., Beijing, China) were housed under pathogen-free (SPF) conditions at 20–25 °C, 55% ± 15% relative humidity, and a 12-h light/dark cycle. Animals had ad libitum access to a standard basal diet (XTI01JX-002, Jiangsu Synergetic Biotechnology Co., Ltd., Nanjing, China) and sterile water. A 5–7-day acclimatization period was observed prior to experimentation. All procedures were approved by the Ethics Committee of Nankai University, China (approval number: 2025-SYDWLL-000505, 1 March 2025).

#### 2.3.2. Acute Toxicity Assessment

Acute oral toxicity was evaluated per China’s national standard GB 15193.3–2014 [23]. Twenty rats (10/sex) were randomized into two groups (5 males and 5 females per group). Following overnight fasting, rats received a single oral gavage of *R*. *eutropha* H16 at 1 × 10^11^ CFU/kg body weight (bw) or sterile water (10 mL/kg bw). Fasting continued for 3–4 h post-administration. Animals were monitored for 24 h for clinical signs of toxicity (mortality, morbidity, and behavioral changes) and observed for 14 days to record their survival and health status.

#### 2.3.3. Subacute Toxicity Evaluation

Subacute toxicity was assessed according to GB 15193.22–2014 [24]. Eighty rats (40/sex) were divided into four groups (10 males and 10 females per group): control (sterile water, 10 mL/kg bw/day), low-dose (1 × 10^8^ CFU/kg bw/day), medium-dose (1 × 10^9^ CFU/kg bw/day), and high-dose (1 × 10^10^ CFU/kg bw/day). Body weight, feed intake, and clinical observations were recorded every 3 days. Terminal analyzes included hematology, serum biochemistry, organ weight measurements, and a histopathological examination of preserved organs (control and high-dose groups only).

#### 2.3.4. Blood and Serum Collection

Under isoflurane anesthesia, blood was collected via retro-orbital phlebotomy. Samples were split into EDTA-K2 anticoagulant tubes for hematological analysis (BC-2800VET, Shenzhen Myriad, Shenzhen, China) within 24 h, and serum separator tubes for biochemical analysis. Serum was obtained by centrifugation (4 °C, 4000× *g*, 15 min) and analyzed using a Pointcare M4 analyzer with Comprehensive I Assay Lyophilized kits (Dymind Biotechnology, Tianjin, China).

#### 2.3.5. Organ Coefficient Calculation

At the final stage of the experiment, the organs of the heart, liver, spleen, kidney, thymus, pancreas, testes of male rats and ovaries of female rats were accurately weighed in all experimental animals. The organ coefficient is calculated using the following formula to standardize the data and eliminate the effect of individual body weight differences:

$$Organ\;coefficient(g/100g)\;=\frac{\mathrm{organ}\;\mathrm{weight}\;\left(g\right)}{\mathrm{body}\;\mathrm{weight}\;\left(g\right)}\times 100$$

#### 2.3.6. Histopathological Analysis

Tissues were fixed in 10% neutral-buffered formalin, dehydrated, and paraffin-embedded. Sections (4~5 μm) were prepared using a LEICA HistoCore MULTICUT microtome, stained with hematoxylin–eosin (H&E), and examined via a digital pathology scanner (Bio-One Scientific Instrument (Beijing) Co., Ltd., Beijing, China) for histopathological changes.

### 2.4. Statistical Analysis

Data are presented as mean ± SD (*n* ≥ 3). One-way ANOVA with post-hoc Tukey’s test was performed using SPSS 25.0. GraphPad Prism 9.5.0 was used for visualization, with significance denoted as follows: * *p* < 0.05, ** *p* < 0.01, *** *p* < 0.001.

## 3. Results and Analysis

### 3.1. Genomic Features and Functional Annotation

**Figure 1:**
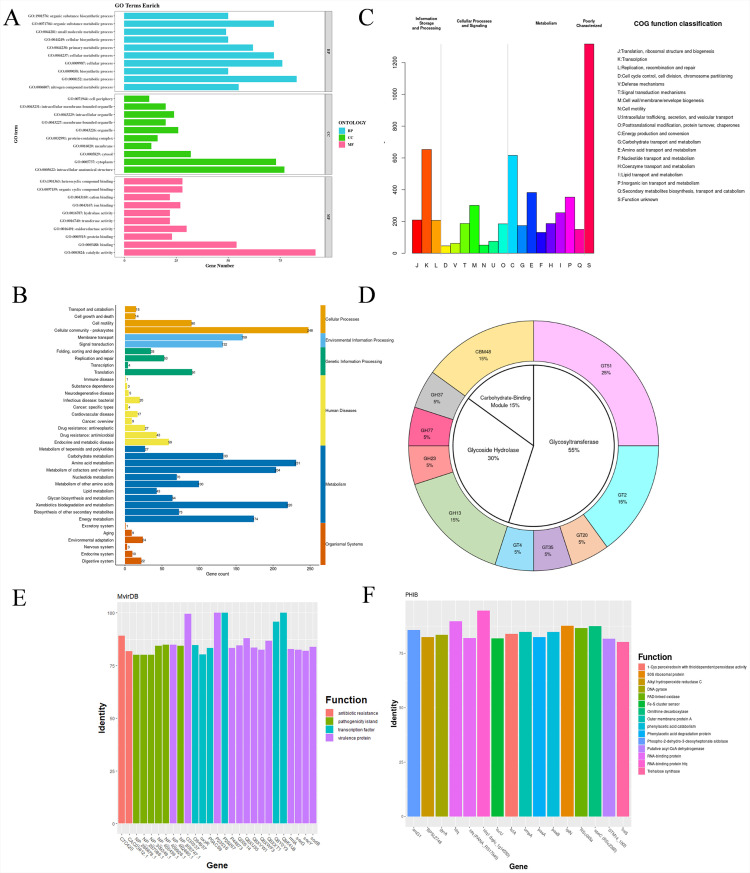
The genomic annotation and analysis of *R.eutropha* H16. (**A**) GO classification statistics. (**B**) KEGG classification statistics. (**C**) COG classification statistics. (**D**) The CAZymes classification of protein functions. (**E**) MirvDB classification statistics. (**F**) PHI-base classification statistics.

The *R*. *eutropha* H16 genome comprises two circular chromosomes and one plasmid (452,139 bp), totaling 7.41 Mbp with 66.5% GC content. Annotation revealed 6807 protein-coding genes spanning 6,550,289 bp (81.4% genome coverage), averaging 962 bp per gene. Gene Ontology (GO) analysis categorized 4082 genes into biological processes (predominantly metabolic regulation and cellular homeostasis), 1111 into molecular functions (primarily catalytic activity and ligand binding), and 569 into cellular components (notably membrane-associated structures and cytoplasmic organelles) (Figure 1A). KEGG pathway mapping assigned 2437 genes to 37 metabolic pathways across six functional categories, with particular enrichment in central carbon metabolism, cellular communication, and stress response systems (Figure 1B). The COG classification of 5541 genes identified transcriptional regulation (11.78%), energy transduction (11.12%), and amino acid transport (6.89%) as predominant functional groups (Figure 1C). CAZy database analysis identified 38 carbohydrate-active enzymes, including 11 glycosyltransferases (GTs), 6 glycoside hydrolases (GHs), and 2 carbohydrate-binding modules (CBMs), representing core carbohydrate metabolism capabilities (Figure 1D). The gene functional profile reveals a robust genetic infrastructure for metabolic versatility, though the limited GH repertoire (six enzymes) suggests a constrained polysaccharide degradation capacity compared to specialized decomposers typically containing >20 GH families.

### 3.2. Virulence and Resistance Analysis

The screening of virulence factors revealed database-dependent discrepancies. VFDB identified two genes linked to immune regulation and effector molecule delivery systems, while MvirDB detected 35 potential virulence genes, and PHI-base identified 25 genes encoding virulence proteins, transcriptional regulators, and virulence island-associated elements (Figure 1C,D); see Supplementary Table S2 for details. Resistance analysis showed no hits in CARD, whereas ARDB identified five mycoplasma peptide resistance-related genes, and ResFam detected two aminoglycoside phosphotransferase-encoding genes (Supplementary Table S3). These discrepancies likely reflect database classification criteria: VFDB focuses on experimentally validated pathogenicity factors, whereas MvirDB employs broader homology-based thresholds. Notably, detected virulence factor genes (e.g., transcriptional regulators) lacked canonical toxin domains, consistent with the strain’s QPS status under EFSA guidelines. Furthermore, the absence of mobile genetic elements (e.g., plasmids and transposons) in resistance loci minimizes horizontal gene transfer risks, a critical safety criterion for food-grade microorganism [25].

### 3.3. Antibiotic Susceptibility Test Results

Antibiotic resistance genes in bacterial genomes pose potential risks if horizontally transferred to pathogens [26]. The genomic analysis of *R*. *eutropha* H16 revealed no mobile genetic elements in resistance loci (Section 3.2), minimizing such risks. To assess biosafety, antibiotic susceptibility was evaluated via the disk diffusion method using antibiotic susceptibility discs. Results were classified per Clinical and Laboratory Standards Institute (CLSI) guidelines. *R*. *eutropha* H16 exhibited resistance to kanamycin, ampicillin, gentamicin, streptomycin, chloramphenicol (9.09 ± 0.49 mm), and tetracycline (10.19 ± 0.87 mm); intermediate resistance to clindamycin (17.03 ± 0.83 mm); and susceptibility to ciprofloxacin (30.49 ± 1.04 mm), erythromycin (34.73 ± 0.83 mm), and vancomycin (25.66 ± 0.33 mm) (Table 1).

### 3.4. The Gastrointestinal Tolerance and Colonization Ability of R. eutropha H16

#### 3.4.1. Tolerance to Low pH, Bile Salts, SGF, and SIF

**Figure 2:**
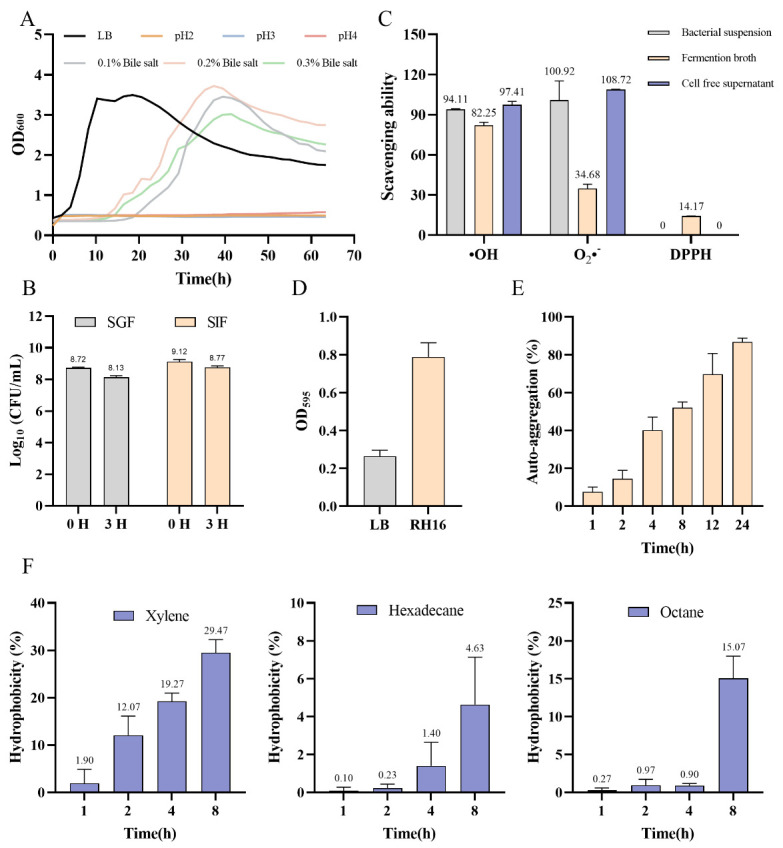
The physiological features analysis of *R. eutropha* H16. (**A**) Growth curves under different acidic conditions (pH = 2, 3, and 4) and different concentrations of bile salts (0.1%, 0.2%, and 0.3%). (**B**) *R. eutropha* H16 viable bacteria at 0 h and 3 h in simulated gastric fluids (SGF) and simulated intestinal fluids (SIF). (**C**) The oxidation resistance of different parts. ·OH, hydroxyl radical U/mL; O_2_^−^, superoxide anion, U/mL; DPPH, 1,1-diphenyl-2-picrylhydrazyl, ug Trolox/mL. (**D**) Biofilm forming capacity. (**E**) Auto-aggregation. (**F**) The hydrophobicity of *R. eutropha* H16 towards xylene, hexadecane, and octane at different time points. “RH16” means *R. eutropha* H16.

Acid and bile salts disrupt bacterial cell membranes and induce DNA damage, leading to protein misfolding and denaturation [25]. To assess gastrointestinal survival potential, *R*. *eutropha* H16 was exposed to acidic conditions (pH 2.0–4.0) and bile salts (0.1–0.3%). Growth curves (Figure 2A) revealed no proliferation under extreme acidity (pH ≤ 4.0) within 48 h, indicating a poor acid tolerance. In contrast, under bile salt stress, the initial growth inhibition (0–12 h) was followed by recovery (12–36 h), with optimal growth at 0.2% bile salts (Figure 2A). This biphasic response suggests a potential activation of bile salt hydrolase (BSH) or efflux systems, enabling metabolic adaptation to sublethal bile concentrations [27]. To further evaluate the gastrointestinal survival, *R*. *eutropha* H16 was exposed to simulated gastric fluid (SGF, pH 2.5) and intestinal fluid (SIF, pH 8.0) for 3 h (Figure 2B). In SGF, viable counts decreased from 8.72 to 8.13 log CFU/mL (25.70% survival), whereas SIF exposure resulted in a smaller decline from 9.12 to 8.77 log CFU/mL (44.67% survival). The higher SIF survival aligns with bile salt tolerance trends, underscoring the strain’s resilience to intestinal conditions despite a limited acid resistance.

#### 3.4.2. Antioxidant Capacity

Metabolic activity generates free radicals and might induce oxidative DNA damage via biomolecular interactions [28]. To characterize the antioxidant potential of *R*. *eutropha* H16, we quantified its radical scavenging activity against three reactive oxygen species: DPPH, hydroxyl radicals, and superoxide anions radicals. As depicted in Figure 2C, the fermentation broth demonstrated a superior DPPH neutralization (14.60 ± 1.24 ug Trolox/mL), while the bacterial suspension and cell-free supernatant exhibited an enhanced hydroxyl radical scavenging (>90 U/mL) and superoxide anion inhibition (>100 U/L), respectively. This compartment-specific antioxidant activity suggests a multifaceted defense strategy, potentially involving extracellular enzymes in the broth and intracellular redox regulators in whole cells, which may collectively mitigate oxidative stress in the gastrointestinal environment.

#### 3.4.3. Surface Adhesion Properties

Biofilm formation represents a survival strategy for bacteria in dynamic environments [29]. *R*. *eutropha* H16’s biofilm-forming capacity was quantified via crystal violet staining. LB medium (OD_595_: 0.2647 ± 0.0302) served as the negative control, yielding an ODc of 0.3554. *R*. *eutropha* H16 (OD_595_ = 0.7881) was classified as a moderate biofilm producer (Figure 2D). Self-aggregation kinetics were monitored over 24 h (Figure 2E). *R*. *eutropha* H16 attained 34.51% self-aggregation at 4 h, 65.08% at 12 h, and 83.66% at 24 h. Per thresholds defined by Montoro et al. [30], aggregation > 50% indicates a high adhesion potential.

Cell surface hydrophobicity, a critical determinant of intestinal epithelial adhesion [31], was solvent-dependent (Figure 2F). *R*. *eutropha* H16 exhibited preferential hydrophobicity toward xylene compared to n-hexadecane and octane (Figure 2F). At 1 h, hydrophobicity in xylene (1.9%) exceeded that of n-hexadecane and octane (<1%). After 8 h, hydrophobicity increased to 29.47% (xylene), 15.07% (octane), and 4.63% (n-hexadecane). Despite temporal increases, hydrophobicity remained lower than probiotic benchmarks, e.g., *Lactobacilli* can reach more than 80% [22]. This solvent selectivity likely reflects variations in surface protein composition and lipid affinity [32,33].

### 3.5. Acute Toxicity Assessment Results

**Figure 3:**
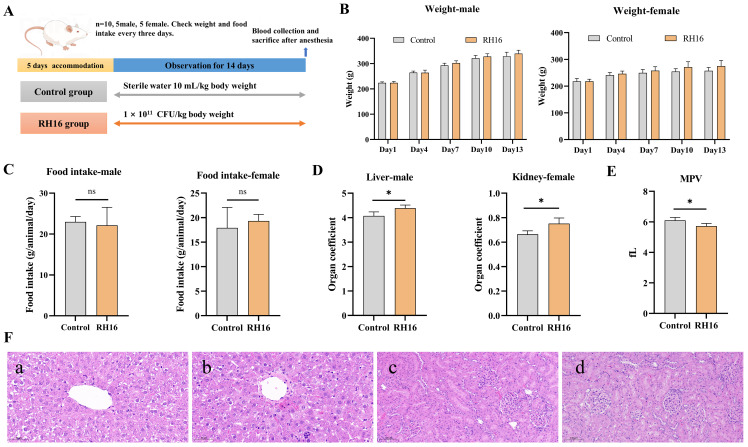
Acute toxicity assessment results of *R. eutropha* H16. (**A**) Acute toxicity experiment design. (**B**) The body weight of male and female rats. (**C**) The food intake of male and female rats. (**D**) The organ coefficient of male rat liver and female rat kidney. (**E**) The MPV value of male rats. MPV mean platelet volume. (**F**) Histopathological results of organs with liver and kidney. a. Liver, male control group, 20×. b. Liver, male RH16 group, 20×. c. Kidney, female control group, 20×. d. Kidney, female control group, 20×. “ns”, no significant difference compared to the control group, *p* > 0.05. * Significantly different from the control group, *p* < 0.05.

To align with OECD guidelines recommending female rats for oral acute toxicity testing due to their higher sensitivity, both sexes were included to comprehensively evaluate *R*. *eutropha* H16’s safety profile (Figure 3A). Throughout the 14-day observation period, no mortality or clinically observable toxicity manifestations (including behavioral alterations, neurological symptoms, or physiological distress) were recorded in any treatment group. The calculated median lethal dose (LD_50_) exceeded 1 × 10^11^ CFU/kg body weight, establishing *R*. *eutropha* H16’s safety profile under acute exposure conditions.

Longitudinal monitoring revealed no statistically significant differences in body weight trajectories (Figure 3B) or feed intake ratios (Figure 3C) between treatment and control cohorts (*p* > 0.05). Postmortem gross pathological examination identified gender-specific alterations in relative organ weights: male subjects exhibited increased hepatic mass indices (*p* < 0.05), while female counterparts demonstrated elevated renal mass indices (*p* < 0.05) (Figure 3D). No other tissue weights were abnormal (Supplementary Table S4). A histopathological investigation of the affected organs via H&E staining revealed focal hepatocellular necrosis in both control and treatment groups (Figure 3F). Notably, lesion characteristics differed between cohorts—control specimens displayed necrotic foci with mononuclear infiltration (Supplemental Figure S1), whereas treatment group lesions showed a reduced necrosis extent (≤5% parenchymal involvement) without inflammatory accompaniment. These findings suggest a spontaneous background pathology unrelated to experimental intervention.

Hematological analysis identified a statistically significant reduction in mean platelet volume (MPV) among male treatment subjects (6.10 ± 0.19 fL vs. 5.72 ± 0.18 fL, *p* < 0.05) (Figure 3E). While platelet activation typically correlates with increased MPV in toxicological responses [34], the observed values remained within the established physiological ranges for Rattus norvegicus (3.8–6.2 fL). No treatment-associated alterations in platelet count (PLT), red cell distribution width (RDW), or other hematological parameters were detected (Supplementary Table S5). The collective findings demonstrate that *R*. *eutropha* H16 administration at 1 × 10^11^ CFU/kg induces no acute toxicological effects, with observed biological variations falling within expected physiological parameters for the model organism.

### 3.6. Subacute Toxicity Evaluation Results

**Figure 4:**
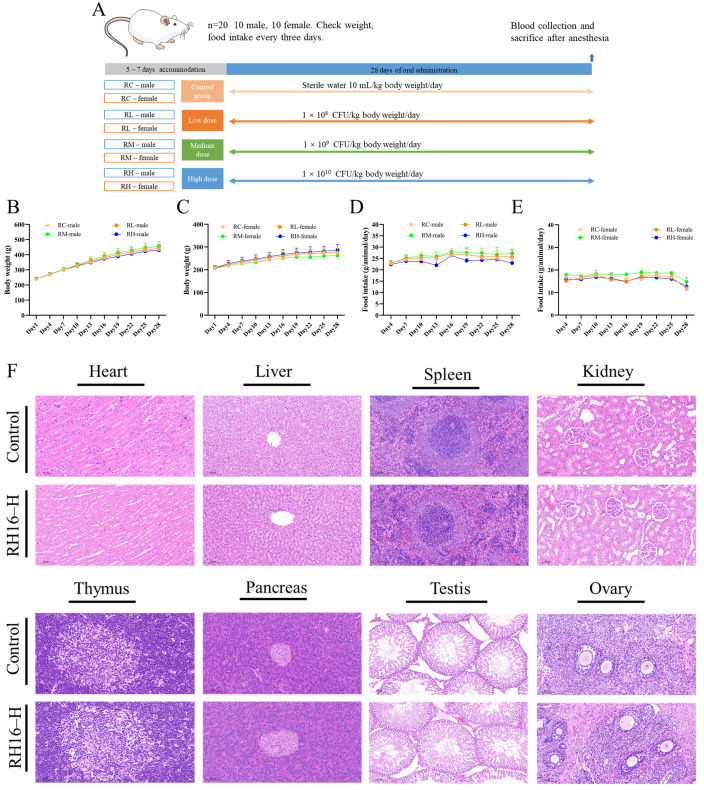
Subacute toxicity assessment results of *R. eutropha* H16. (**A**) Subacute toxicity experiment design. (**B**) Body weight of male. (**C**) Body weight of female. (**D**) Food intake of male. (**E**) Food intake of female. RC control group; RL *R. eutropha* H16 low-dose, 10^8^ CFU/kg/day; RM *R. eutropha* H16 medium-dose, 10^9^ CFU/kg/day; RH *R. eutropha* H16 high-dose, 10^10^ CFU/kg/day. (**F**) The histopathological results of the control group and high-dose group. Heart, liver, spleen, kidney, pancreas, testis, ovary, 20×; thymus, 40×. RH16-H, *R. eutropha* H16 high-dose group.

Based on the acute toxicity study results (LD₅₀ > 1 × 10^11^ CFU/kg bw), a 28-day repeated-dose oral toxicity study was conducted to assess *R*. *eutropha* H16’s subchronic toxicity risk. Doses of 1 × 10^8^, 1 × 10^9^, and 1 × 10^10^ CFU/kg/day (denoted RL, RM, RH) were administered to rats (Figure 4A), enabling the systematic evaluation of safety margins and dose-dependent effects. Body weight and food consumption were recorded every three days. No significant differences in weight gain or food intake were observed between experimental and control groups (Figure 4B–E), indicating no adverse effects on growth or appetite at tested doses.

Hematological parameters showed no treatment-related alterations across groups (Table 2). Serum biochemistry revealed no abnormalities in female rats (*p* > 0.05). In males, however, the RH group exhibited significantly decreased aspartate aminotransferase (AST: 282.0 ± 70.28 vs. 179.5 ± 60.25 U/L, *p* < 0.05), while the RM group showed elevated globulin (GLO: 41.17 ± 4.88 vs. 48.95 ± 6.95 g/L) and high-density lipoprotein cholesterol (HDL-C: 1.05 ± 0.41 vs. 1.62 ± 0.23 mmol/L, *p* < 0.05) (Table 3). Notably, elevated globulin levels may indicate enhanced immunoglobulin production rather than hepatic dysfunction [35]. The cardioprotective HDL-C increase [35] and clinically insignificant AST reduction [36] suggest adaptive metabolic responses rather than pathological effects.

Organ coefficients analysis identified a modest reduction of pancreatic coefficients of high-dose females (0.23 ± 0.02 vs. 0.19 ± 0.03, *p* < 0.05), while other organs showed no significant mass alterations (Table 4). Histopathological examination confirmed structural integrity across all evaluated tissues (Figure 4F). Pancreatic lobules maintained distinct exocrine–acinar architecture with normal islet cell distribution, demonstrating a preserved endocrine function. No evidence of necrosis, inflammatory infiltration, or fibrotic remodeling was observed in other organs. These collective findings demonstrate that prolonged *R*. *eutropha* H16 exposure induces no dose-dependent toxicological effects, with observed biochemical fluctuations remaining within physiological adaptation thresholds.

## 4. Discussion

The expanding utilization of microbial platforms in food systems necessitates comprehensive safety frameworks that integrate genomic characterization with functional toxicology [37]. Our multi-parametric assessment of *R*. *eutropha* H16 advances this paradigm by reconciling genotypic predictions with phenotypic validation, establishing critical benchmarks for industrial microbe safety evaluation.

While whole-genome analysis confirmed the absence of canonical virulence determinants, the observed discordance between resistance genotypes and phenotypes underscores a critical limitation of purely computational risk assessment. This aligns with Rasheed’s demonstration that single resistance markers poorly predict phenotypic susceptibility [38]. For aminoglycoside antibiotic resistance, the APH3 gene, which encodes an aminoglycoside phosphotransferase that modifies kanamycin, gentamicin, and streptomycin by phosphorylation, rendering them incapable of binding to their ribosomal targets and causing resistance, was detected in the Resfams database [39]. The high coverage and concordance values indicate a functional homologous gene, identifying a direct link between the resistance gene and the phenotype. As for antibiotic resistance where no specific genes were detected, such as ampicillin, chloramphenicol, tetracycline, clindamycin, and other resistance phenotypes, it is hypothesized that there are diverse mechanisms of resistance. Researchers have now identified a variety of factors associated with antibiotic resistance, including mutations in antibiotic targets or transporter proteins, mutations in regulators of resistance genes, an increased expression of genes encoding efflux pumps, and a decreased permeability of the outer membrane [40]. For example, the efflux pump actively excretes a wide range of antibiotics from the cell, reducing intracellular concentrations [41]. Altered outer membrane pore proteins reduce antibiotic permeability, especially to hydrophilic antibiotics [42]. The presence of a single resistance gene may not be sufficient to fully explain the resistance phenotype, and synergistic effects of multiple resistance mechanisms need to be considered [43]. In addition, *R*. *eutropha* may have inherent intrinsic resistance mechanisms that act synergistically with the above mechanisms. Li et al. on photodynamic therapy to combat drug-resistant bacteria showed a 64-fold reduction in chloramphenicol-resistant concentrations through a bifunctional photosensitizer, OPFV-NB, which resulted in the inhibition of efflux pump activity [44]. The mechanisms of antibiotic resistance are complex, and future studies are necessary to develop comprehensive assessment programs such as efflux pump modulation and membrane permeability modulation to further explore the causes of strain resistance. Vougiouklaki conducted a tolerance study on four standard lactic acid bacteria, *Lactobacillus gasseri*, *Lactiplantibacillus plantarum*, *Lacticaseibacillus rhamnosus* GG, and *Levilactobacillus brevis*, and the results showed that after 3 h in SGF, the survival rate of all four lactic acid bacteria was above 98%. In contrast, the survival rate of *L*. *gasseri* D was only 49.43% after 3 h in SIF, and the survival rates of the remaining three strains of lactic acid bacteria were all above 80%, although they decreased compared with those in SGF [45]. In contrast, *R*. *eutropha* H16 had a lower survival rate in the simulated gastrointestinal environment, and its survival was very limited. However, it is worth noting that. *R*. *eutropha* H16’s robust tolerance to bile salts (0.2% optimal growth) suggests functional bile salt hydrolase (BSH) activity, a trait critical for intestinal survival and cholesterol-lowering effects [21]. However, when exposed to bile salts under similar conditions, the survival rate of *L*. *rhamnosus* decreased from 10.87 ± 0.5 log CFU/mL to 3.0 ± 0.4 log CFU/mL (−7.87 log) [46]. The BSH-mediated deconjugation of bile acids not only disrupts cholesterol absorption but also enhances fecal excretion, a mechanism validated in probiotics like *Lactobacillus* spp. [47,48]. However, unlike well-characterized probiotics, *R*. *eutropha* H16’s BSH pathway and its interplay with metabolic networks remain unexplored. Future studies should employ transcriptomics to map bile-responsive gene networks, which could unveil novel targets for engineering strains with enhanced hypocholesterolemic properties.

The colonization potential of strain *R*. *eutropha* H16 in the intestine was assessed via biofilm formation, hydrophobicity, and auto-aggregation. RH16 showed a moderate biofilm-forming ability—a high auto-aggregation (83.66%) but low hydrophobicity (29.47% in xylene). In comparison to other probiotics, the hydrophobicity of *Lactobacillus paracasei* ranged from 24.16% to 68.18%, and *Lactobacillus plantarum* was hydrophobic to n-hexadecane up to 70% [49]. This suggests that its colonization may depend on bacterial interactions rather than host cell adhesion. While the correlation between surface properties and colonization efficiency remains debated, current research indicates that strains with a high surface hydrophobicity and strong self-aggregation are more likely to effectively bind human intestinal cells [21]. Future research can also further investigate its adhesion properties to intestinal epithelial cells by detecting their adhesion characteristics. The strain’s biofilm proficiency—while enhancing ecological competitiveness [50]—warrants surveillance given the biofilm-associated AMR amplification observed in *Salmonella* (60% multidrug resistance correlation) [51].

The absence of mortality or overt toxicity across acute and subacute exposures confirms the baseline safety of *R*. *eutropha* H16, while biochemical modulations reveal potential therapeutic synergies. HDL-C, a key lipid carrier facilitating reverse cholesterol transport and atherosclerotic plaque inhibition, demonstrates cardioprotective effects with a 2–3% coronary risk reduction per 1 mg/dL increment [52]. Furthermore, the reduction in AST may be consistent with the dietary intervention of Hakkak [53] and Herrera et al. [54] in an animal model of obesity, reflecting an improvement in hepatic lipid metabolism after an intervention through healthy foods. Therefore, the slight changes in serum are not only unrelated to toxic effects but also may demonstrate the potential probiotic effects of *R*. *eutropha* H16 on animals.

In addition, changes in organ coefficients have also attracted our focused attention. Some researchers proposed that organ indices reflect animal development and function, correlating positively with relative organ weight. Other researchers posit that abnormal coefficients indicate pathological changes—elevated values suggesting congestion/edema/hypertrophy, while reduced values implying atrophy/degeneration. While acute exposure induced gender-specific mass fluctuations (males’ liver +18%, females’ kidney +15%), histopathological integrity and functional markers support Schauss’s paradigm that weight changes require pathological confirmation [55]. Combined with normal serum glucose levels, pancreatic functionality remained unaffected, and the pancreatic coefficients of high-dose females reduction (0.23 → 0.19) without architectural disruption (Figure 4F) likely reflects physiological adaptation rather than toxicity, consistent with the nutrient-sensing modulation observed in *L*. *barbarum* interventions [56]. Collectively, toxicological evaluations indicate that *R*. *eutropha* H16 exhibited excellent tolerance and safety across all tested doses. This systematic safety framework establishes *R*. *eutropha* H16 as a viable food-grade chassis while providing methodological blueprints for next-generation microbial risk assessment.

## 5. Conclusions

This study establishes *R*. *eutropha* H16 as a biosafe candidate for food applications through genomic, phenotypic, and toxicological analyzes. Genomic screening confirmed minimal virulence factors and antibiotic resistance genes, aligning with its QPS status and reinforcing its non-pathogenicity. Phenotypic assays revealed a robust bile salt tolerance (0.2% optimal growth) and moderate gastrointestinal survival, though the low hydrophobicity (29.47% in xylene) suggests colonization may depend on microbial interactions rather than host adhesion, contrasting with high-hydrophobicity probiotics like *L*. *plantarum*. Toxicological evaluations in rodents showed no mortality or pathology, with organ weight and serum changes (e.g., elevated HDL-C) interpreted as adaptive responses, consistent with Schauss’s emphasis on histopathological validation [55]. This work bridges genomic data with phenotypic outcomes, offering a framework for strain-level safety validation in food biotechnology. Chronic toxicity data and mechanistic insights into antioxidant/cholesterol pathways remain unexplored. Future research should employ transcriptomics to resolve BSH networks and assess long-term safety in germ-free models. Additionally, adaptive evolution could enhance hydrophobicity or probiotic efficacy. This study advances *R*. *eutropha* H16’s safety profile while underscoring opportunities for biotechnological innovation. Future work could assess the impact on chronic toxic exposure in depth and conduct mechanistic studies on strain-specific antioxidants and cholesterol-modulating properties to expand its potential for food applications.

## Figures and Tables

**Table 1:** Antibiotic susceptibility.

Antibiotic	Concentration (μg/Piece)	Inhibition Zone (mm)	Sensitivity
Kanamycin	30	0	R
Ampicillin	10	0	R
Gentamicin	10	0	R
Streptomycin	10	0	R
Chloramphenicol	30	9.09 ± 0.49	R
Tetracycline	30	10.19 ± 0.87	R
Clindamycin	2	17.03 ± 0.83	I
Ciprofloxacin	5	30.49 ± 1.04	S
Erythromycin	15	34.73 ± 0.83	S
Vancomycin	30	25.66 ± 0.33	S

“R” indicates resistant; “I” indicates moderately sensitive; “S” indicates sensitive.

**Table 2:** Hematological analysis results of subacute toxicity.

Parameters	Control-Male	RL-Male	RM-Male	RH-Male	Control-Female	RL-Female	RM-Female	RH-Female
WBC (10^9^/L)	12.84 ± 2.28	10.88 ± 2.81	11.08 ± 2.12	11.96 ± 3.09	9.16 ± 2.5	9.78 ± 1.67	9.81 ± 2.29	10.46 ± 2.54
Lymph (10^9^/L)	9.95 ± 1.57	8.87 ± 2.05	8.79 ± 1.37	9.28 ± 1.95	7.50 ± 2.02	7.88 ± 1.49	7.69 ± 2.11	8.04 ± 2.33
Mon (10^9^/L)	0.36 ± 0.14	0.25 ± 0.11	0.25 ± 0.08	0.26 ± 0.16	0.18 ± 0.06	0.23 ± 0.05	0.24 ± 0.07	0.22 ± 0.08
Gran (10^9^/L)	2.53 ± 0.86	1.76 ± 0.85	2.04 ± 1.39	2.42 ± 1.73	1.48 ± 0.54	1.67 ± 0.48	1.88 ± 0.68	2.2 ± 1.48
Lymph% (%)	77.77 ± 4.74	82.03 ± 4.48	80.03 ± 7.95	78.71 ± 9.34	81.95 ± 2.83	80.54 ± 4.26	77.92 ± 6.71	77.27 ± 11.5
Mon% (%)	2.85 ± 0.52	2.33 ± 0.47	2.31 ± 0.44	2.38 ± 0.71	2.05 ± 0.35	2.24 ± 0.30	2.49 ± 0.48	2.20 ± 0.50
Gran% (%)	19.38 ± 4.41	15.64 ± 4.06	17.66 ± 7.82	18.91 ± 8.71	16 ± 2.74	17.22 ± 4.14	19.59 ± 6.42	20.53 ± 11.29
RBC (10^12^/L)	7.58 ± 0.33	7.69 ± 0.51	7.34 ± 0.24	7.38 ± 0.49	7.39 ± 0.33	7.42 ± 0.21	7.38 ± 0.36	7.34 ± 0.36
HGB (g/L)	148.2 ± 8.0	149.1 ± 7.4	144.8 ± 3.39	143.3 ± 10.38	143.0 ± 8.67	142.7 ± 3.50	141.6 ± 6.47	140.4 ± 6.26
HCT (%)	46.78 ± 2.54	46.85 ± 2.68	45.99 ± 1.28	45.81 ± 2.64	44.79 ± 2.87	44.43 ± 1.22	44.6 ± 1.77	44.41 ± 2.26
MCV (fL)	61.8 ± 2.21	61.0 ± 1.79	62.78 ± 1.86	62.21 ± 1.88	60.63 ± 2.11	60.02 ± 1.94	60.58 ± 1.02	60.6 ± 1.73
MCH (pg)	19.52 ± 0.9	19.35 ± 0.61	19.70 ± 0.54	19.39 ± 0.85	19.28 ± 0.44	19.2 ± 0.45	19.15 ± 0.49	19.10 ± 0.48
MCHC (g/L)	316.3 ± 6.8	317.8 ± 3.99	314.2 ± 4.89	312.1 ± 7.17	318.9 ± 5.59	320.7 ± 5.19	316.8 ± 4.08	315.8 ± 8.09
RDW (%)	11.21 ± 0.54	11.33 ± 0.38	11.43 ± 0.52	11.35 ± 0.53	10.31 ± 0.2	10.62 ± 0.36	10.54 ± 0.53	10.79 ± 0.57
PLT (10^9^/L)	1305.5 ± 166.06	1229.6 ± 149.34	1084.9 ± 190.57	1130.8 ± 224.75	1212.3 ± 210.49	1303.5 ± 178.61	1223.5 ± 112.56	1136.6 ± 223.02
MPV (fL)	6.14 ± 0.20	6.11 ± 0.40	5.91 ± 0.27	5.99 ± 0.31	5.96 ± 0.34	5.78 ± 0.2	5.72 ± 0.26	5.84 ± 0.24
PDW	16.13 ± 0.18	16.22 ± 0.19	16.27 ± 0.25	16.23 ± 0.16	15.92 ± 0.17	16 ± 0.11	15.99 ± 0.19	16.11 ± 0.19
PCT (%)	0.66 ± 0.04	0.63 ± 0.02	0.61 ± 0.06	0.6 ± 0.06	0.64 ± 0.07	0.65 ± 0.03	0.64 ± 0.04	0.61 ± 0.10

Results are expressed as mean ± SD (male/female *n* = 10). WBC white blood cell count; Lymph Lymphocyte count; Mon monocyte count; Gran neutrophil count; Lymph% lymphocyte percentage; Mon% monocyte percentage; Gran% neutrophil percentage; RBC red blood cell count; HGB hemoglobin; HCT erythrocyte pressure volume; MCV mean erythrocyte volume; MCH mean erythrocyte hemoglobin content; MCHC mean erythrocyte hemoglobin concentration; RDW coefficient of variation of red cell distribution width; PLT platelet number; MPV mean platelet volume; PDW platelet distribution width; PCT Plateletcrit.

**Table 3:** Serum biochemistry analysis results of subacute toxicity.

Parameters	Control-Male	RL-Male	RM-Male	RH-Male	Control-Female	RL-Female	RM-Female	RH-Female
ALT (U/L)	44.29 ± 6.8	50.22 ± 13.74	48.3 ± 8.88	47.6 ± 9.7	57.1 ± 16.58	53.8 ± 6.01	51.56 ± 12.95	46.44 ± 9.89
AST (U/L)	282 ± 70.28	230.89 ± 91.12	242.3 ± 87.5	179.5 ± 60.25 *	271.6 ± 69.15	243.3 ± 68.26	248.33 ± 38.33	209.67 ± 61.86
TBIL (μmol/L)	4.59 ± 1.53	4.88 ± 1.95	5.40 ± 3.25	3.98 ± 1.71	3.58 ± 1.55	3.36 ± 0.92	3.66 ± 1.11	2.93 ± 0.71
IBIL (μmol/L)	4.40 ± 1.45	4.29 ± 1.68	4.88 ± 2.87	3.30 ± 1.61	3.25 ± 1.5	2.93 ± 0.66	3.12 ± 1.27	2.46 ± 0.88
TP (g/L)	65.31 ± 7.59	68.59 ± 7.32	75.79 ± 10.94	74.43 ± 8.45	86.25 ± 6.82	87.98 ± 12.23	84.94 ± 12.12	83.74 ± 9.68
ALB (g/L)	24.14 ± 2.98	24.89 ± 2.72	26.75 ± 8.96	26.99 ± 3.14	40.13 ± 5.56	41.53 ± 10.64	33.86 ± 13.45	31.8 ± 9.84
GLO (g/L)	41.17 ± 4.88	43.38 ± 4.65	48.95 ± 6.95 *	47.62 ± 5.77	48.8 ± 2.98	48.54 ± 7.3	55.11 ± 12.62	52.11 ± 18.57
UREA (mmol/L)	4.79 ± 0.38	5.42 ± 1.38	5.96 ± 0.85	5.46 ± 0.89	7.68 ± 1.84	7.89 ± 1.61	7.02 ± 1.48	6.75 ± 0.83
CRE (μmol/L)	45.57 ± 6.50	47.22 ± 10.34	50.40 ± 8.13	47.1 ± 5.63	67.0 ± 8.89	65.8 ± 8.89	62.89 ± 6.21	58.78 ± 7.64
GLU (mmol/L)	8.06 ± 1.45	11.71 ± 4.19	11.40 ± 2.56	11.56 ± 2.35	13.01 ± 4.72	11.74 ± 2.58	10.7 ± 1.31	11.33 ± 2.39
TG (mmol/L)	3.36 ± 0.96	3.03 ± 1.57	3.28 ± 1.56	2.87 ± 1.47	1.99 ± 0.66	1.88 ± 0.30	1.83 ± 0.49	1.57 ± 0.37
CHOL (mmol/L)	2.61 ± 0.86	3.13 ± 0.74	3.19 ± 0.39	2.86 ± 0.42	3.20 ± 0.27	3.30 ± 0.41	2.97 ± 0.26	3.03 ± 0.42
HDL-C (mmol/L)	1.05 ± 0.41	1.54 ± 0.48	1.62 ± 0.23 *	1.42 ± 0.37	1.95 ± 0.44	2.12 ± 0.49	1.62 ± 0.21	1.61 ± 0.51
LDL-C (mmol/L)	0.33 ± 0.22	0.47 ± 0.28	0.28 ± 0.19	0.41 ± 0.24	0.58 ± 0.22	0.49 ± 0.13	0.58 ± 0.1	0.59 ± 0.19

Results are expressed as mean ± SD (male/female *n* = 10). * Significantly different from the control group, *p* < 0.05. ALT alanine aminotransferase; AST aspartate aminotransferase; TBIL total bilirubin; IBIL indirect bilirubin; TP total protein; ALB Albumin; GLO globulin; UREA urea; CRE Creatinine; GLU glucose; TG Triglycerides; CHOL cholesterol; HDL-C high-density lipoprotein cholesterol; LDL-C low-density lipoprotein cholesterol.

**Table 4:** Organ coefficients of subacute toxicity.

Parameters	RC-Male	RL-Male	RM-Male	RH-Male	RC-Female	RL-Female	RM-Female	RH-Female
Heart	0.38 ± 0.06	0.42 ± 0.09	0.34 ± 0.02	0.35 ± 0.05	0.37 ± 0.04	0.4 ± 0.06	0.38 ± 0.05	0.36 ± 0.04
Thymus	0.17 ± 0.03	0.17 ± 0.03	0.17 ± 0.02	0.17 ± 0.03	0.20 ± 0.04	0.20 ± 0.04	0.18 ± 0.04	0.20 ± 0.04
Liver	3.98 ± 0.37	3.99 ± 0.33	4.11 ± 0.23	3.95 ± 0.44	3.45 ± 0.22	3.50 ± 0.26	3.39 ± 0.29	3.52 ± 0.27
Spleen	0.18 ± 0.02	0.17 ± 0.03	0.19 ± 0.02	0.18 ± 0.02	0.22 ± 0.03	0.23 ± 0.04	0.23 ± 0.05	0.21 ± 0.03
Pancreas	0.18 ± 0.04	0.21 ± 0.04	0.19 ± 0.03	0.20 ± 0.04	0.23 ± 0.02	0.22 ± 0.04	0.21 ± 0.04	0.19 ± 0.03 *
Kidney	0.73 ± 0.05	0.70 ± 0.08	0.72 ± 0.05	0.74 ± 0.04	0.59 ± 0.04	0.66 ± 0.13	0.65 ± 0.07	0.65 ± 0.08
Testis	0.79 ± 0.05	0.80 ± 0.06	0.77 ± 0.06	0.76 ± 0.09	—	—	—	—
Ovary	—	—	—	—	0.05 ± 0.01	0.06 ± 0.01	0.06 ± 0.01	0.06 ± 0.01

Results are expressed as mean ± SD (male/female *n* = 10). * Significantly different from the control group, *p* < 0.05.

## Data Availability

The original contributions presented in this study are included in the article/Supplementary Material. Further inquiries can be directed to the corresponding authors.

## Supplementary Materials

The following supporting information can be downloaded at: https://www.mdpi.com/article/10.3390/microorganisms13061323/s1, Table S1: Criteria for determination of drug sensitive paper. Figure S1: Histopathological of liver with acute toxicity control group. Necrotic area of liver cells (black circle), necrotic liver cells (long tail arrow), infiltration of monocytes (no tail arrow), 40×. Table S2: List of virulence factor identified in the *Ralstonia eutropha* H16 genome. Table S3: List of antibiotic resistance genes identified in the *Ralstonia eutropha* H16 genome. Table S4: Organ coefficients of acute toxicity. Table S5: Hematological result of acute toxicity.

## References

[1] Jiang Y., Yang X., Zeng D., Su Y., Zhang Y. (2022). Microbial conversion of syngas to single cell protein: The role of carbon monoxide. Chem. Eng. J..

[2] Pohlmann A., Fricke W.F., Reinecke F., Kusian B., Liesegang H., Cramm R., Eitinger T., Ewering C., Pötter M., Schwartz E. (2006). Genome sequence of the bioplastic-producing “Knallgas” bacterium *Ralstonia eutropha* H16. Nat. Biotechnol..

[3] Hanko E.K.R., Sherlock G., Minton N.P., Malys N. (2022). Biosensor-informed engineering of *Cupriavidus necator* H16 for autotrophic D-mannitol production. Metab. Eng..

[4] Raberg M., Volodina E., Lin K., Steinbüchel A. (2017). *Ralstonia eutropha* H16 in progress: Applications beside PHAs and establishment as production platform by advanced genetic tools. Crit. Rev. Biotechnol..

[5] Lee Y.J., Moon B.C., Lee D.K., Ahn J.H., Gong G., Um Y., Lee S.-M., Kim K.H., Ko J.K. (2025). Sustainable production of microbial protein from carbon dioxide in the integrated bioelectrochemical system using recycled nitrogen sources. Water Res..

[6] Yu J. (2018). Fixation of carbon dioxide by a hydrogen-oxidizing bacterium for value-added products. World J. Microbiol. Biotechnol..

[7] Li Y.P., Ahmadi F., Kariman K., Lackner M. (2024). Recent advances and challenges in single cell protein (SCP) technologies for food and feed production. NPJ Sci. Food.

[8] Li R., Jiang Y., Huang J., Luo K., Fan X., Guo R., Liu T., Zhang Y., Fu S. (2024). Simultaneous biogas upgrading and single cell protein production using hydrogen oxidizing bacteria. Chem. Eng. J..

[9] Wang L., Yao J., Tu T., Yao B., Zhang J. (2024). Heterotrophic and autotrophic production of L-isoleucine and L-valine by engineered *Cupriavidus necator* H16. Bioresour. Technol..

[10] Tang R., Xu R., Gao X., Dai C., Qin X., Yang J. (2025). Production of α-amylase from gluconate and carbon dioxide by protein synthesis and secretion optimization in *Cupriavidus necator* H16. Bioresour. Technol..

[11] Lin Y.-C., Ng I.S. (2025). Biofabrication of polyhydroxybutyrate (PHB) in engineered *Cupriavidus necator* H16 from waste molasses. J. Taiwan. Inst. Chem. Eng..

[12] Sirohi R., Prakash Pandey J., Kumar Gaur V., Gnansounou E., Sindhu R. (2020). Critical overview of biomass feedstocks as sustainable substrates for the production of polyhydroxybutyrate (PHB). Bioresour. Technol..

[13] Koutsoumanis K., Allende A., Alvarez-Ordóñez A., Bolton D., Bover-Cid S., Chemaly M., Davies R., De Cesare A., Hilbert F., Lindqvist R. (2020). Update of the list of QPS-recommended biological agents intentionally added to food or feed as notified to EFSA 11: Suitability of taxonomic units notified to EFSA until September 2019. EFSA J..

[14] Pradhan D., Mallappa R.H., Grover S. (2020). Comprehensive approaches for assessing the safety of probiotic bacteria. Food Control.

[15] Liu H., Ma J., Yang P., Geng F., Li X., Lü J., Wang Y. (2024). Comparative analysis of biofilm characterization of probiotic *Escherichia coli*. Front. Microbiol..

[16] Peng Y.-Y., Zhong S.-Y., Xu X.-L., Liu D.-M. (2023). Analysis of the safety and probiotic properties of *Bifidobacterium longum* B2-01 by complete genome sequencing combined with corresponding phenotypes. LWT.

[17] Zhang C., Ma K., Nie K., Deng M., Luo W., Wu X., Huang Y., Wang X. (2022). Assessment of the safety and probiotic properties of *Roseburia intestinalis*: A potential “Next Generation Probiotic”. Front. Microbiol..

[18] Feng S., Wang H., Lin X., Liang H., Zhang S., Chen Y., Ji C. (2023). Probiotic properties of *Lactobacillus plantarum* and application in prebiotic gummies. LWT.

[19] Gu X., Wang H., Wang L., Zhang K., Tian Y., Wang X., Xu G., Guo Z., Ahmad S., Egide H. (2024). The antioxidant activity and metabolomic analysis of the supernatant of *Streptococcus alactolyticus* strain FGM. Sci. Rep..

[20] Cozzolino A., Vergalito F., Tremonte P., Iorizzo M., Lombardi S.J., Sorrentino E., Luongo D., Coppola R., Di Marco R., Succi M. (2020). Preliminary Evaluation of the Safety and Probiotic Potential of *Akkermansia muciniphila* DSM 22959 in Comparison with Lactobacillus rhamnosus GG. Microorganisms.

[21] Alizadeh Behbahani B., Jooyandeh H., Hojjati M., Ghodsi Sheikhjan M. (2024). Evaluation of probiotic, safety, and anti-pathogenic properties of *Levilactobacillus brevis* HL6, and its potential application as bio-preservatives in peach juice. LWT.

[22] Tarique M., Abdalla A., Masad R., Al-Sbiei A., Kizhakkayil J., Osaili T., Olaimat A., Liu S.-Q., Fernandez-Cabezudo M., al-Ramadi B. (2022). Potential probiotics and postbiotic characteristics including immunomodulatory effects of lactic acid bacteria isolated from traditional yogurt-like products. LWT.

[23] (2014). National Food Safety Standard—Acute Toxicity Test.

[24] (2014). National Food Safety Standard—28 Days Oral Toxicity Test.

[25] Papadimitriou K., Alegría Á., Bron Peter A., de Angelis M., Gobbetti M., Kleerebezem M., Lemos José A., Linares Daniel M., Ross P., Stanton C. (2016). Stress Physiology of Lactic Acid Bacteria. Microbiol. Mol. Biol. Rev..

[26] Saboktakin-Rizi M., Alizadeh Behbahani B., Hojjati M., Noshad M. (2021). Identification of *Lactobacillus plantarum* TW29-1 isolated from Iranian fermented cereal-dairy product (Yellow Zabol Kashk): Probiotic characteristics, antimicrobial activity and safety evaluation. J. Food Meas. Charact..

[27] Lou H., Wang J., Wang Y., Gao Y., Wang W. (2024). Comprehensive assessment of *Enterococcus faecalis* SN21-3: Probiotic features and safety evaluation for potential animal use. Food Biosci..

[28] Cong S., Zhang X., Ji J., Liu X., Hu N. (2024). Isolation and identification of blueberry-derived lactic acid bacteria and their probiotic, antioxidant, and fermentation properties. Food Biosci..

[29] Flemming H.-C., Wingender J., Szewzyk U., Steinberg P., Rice S.A., Kjelleberg S. (2016). Biofilms: An emergent form of bacterial life. Nat. Rev. Microbiol..

[30] Montoro B.P., Benomar N., Lavilla Lerma L., Castillo Gutiérrez S., Gálvez A., Abriouel H. (2016). Fermented Aloreña Table Olives as a Source of Potential Probiotic *Lactobacillus pentosus* Strains. Front. Microbiol..

[31] Maione A., Imparato M., Buonanno A., Salvatore M.M., Carraturo F., de Alteriis E., Guida M., Galdiero E. (2024). Evaluation of Potential Probiotic Properties and In Vivo Safety of Lactic Acid Bacteria and Yeast Strains Isolated from Traditional Home-Made Kefir. Foods.

[32] Chantanawilas P., Pahumunto N., Teanpaisan R. (2024). Aggregation and adhesion ability of various probiotic strains and Candida species: An in vitro study. J. Dent. Sci..

[33] Rocha-Mendoza D., Kosmerl E., Miyagusuku-Cruzado G., Giusti M.M., Jiménez-Flores R., García-Cano I. (2020). Growth of lactic acid bacteria in milk phospholipids enhances their adhesion to Caco-2 cells. J. Dairy Sci..

[34] Kumar V., Stewart Iv J.H. (2024). Platelet’s plea to Immunologists: Please do not forget me. Int. Immunopharmacol..

[35] Abdel-Tawwab M., Eissa E.-S.H., Tawfik W.A., Abd Elnabi H.E., Saadony S., Bazina W.K., Ahmed R.A. (2022). Dietary curcumin nanoparticles promoted the performance, antioxidant activity, and humoral immunity, and modulated the hepatic and intestinal histology of Nile tilapia fingerlings. Fish Physiol. Biochem..

[36] Tamber S.S., Bansal P., Sharma S., Singh R.B., Sharma R. (2023). Biomarkers of liver diseases. Mol. Biol. Rep..

[37] Rychen G., Aquilina G., Azimonti G., Bampidis V., Bastos M.d.L., Bories G., Chesson A., Cocconcelli P.S., Flachowsky G., Gropp J. (2018). Guidance on the characterisation of microorganisms used as feed additives or as production organisms. EFSA J..

[38] Rasheed H., Ijaz M., Ahmed A., Javed M.U., Shah S.F.A., Anwaar F. (2023). Discrepancies between phenotypic and genotypic identification methods of antibiotic resistant genes harboring Staphylococcus aureus. Microb. Pathog..

[39] Kaplan E., Chaloin L., Guichou J.-F., Berrou K., Rahimova R., Labesse G., Lionne C. (2025). APH Inhibitors that Reverse Aminoglycoside Resistance in *Enterococcus casseliflavus*. ChemMedChem.

[40] Song D., Jia A., Liu B., Liu S., Dong K., Man C., Yang X., Jiang Y. (2023). Whole-transcriptome analysis after the acquisition of antibiotic resistance of *Cronobacter sakazakii*: Mechanisms of antibiotic resistance and virulence changes. Food Res. Int..

[41] Chen Q., Gong X., Zheng F., Ji G., Li S., Stipkovits L., Szathmary S., Liu Y. (2018). Interplay Between the Phenotype and Genotype, and Efflux Pumps in Drug-Resistant Strains of *Riemerella anatipestifer*. Front. Microbiol..

[42] Ganjo A.R., Balaky S.T.J., Mawlood A.H., Smail S.B., Shabila N.P. (2024). Characterization of genes related to the efflux pump and porin in multidrug-resistant Escherichia coli strains isolated from patients with COVID-19 after secondary infection. BMC Microbiol..

[43] Sparbrod M., Gager Y., Koehler A.-K., Jentsch H., Stingu C.-S. (2023). Relationship between Phenotypic and Genotypic Resistance of Subgingival Biofilm Samples in Patients with Periodontitis. Antibiotics.

[44] Li M., Li L., Zhang X., Yuan Q., Bao B., Tang Y. (2025). A Conjugated Oligomer with Drug Efflux Pump Inhibition and Photodynamic Therapy for Synergistically Combating Resistant Bacteria. ACS Appl. Mater. Interfaces.

[45] Vougiouklaki D., Tsironi T., Tsantes A.G., Tsakali E., Van Impe J.F.M., Houhoula D. (2023). Probiotic Properties and Antioxidant Activity In Vitro of Lactic Acid Bacteria. Microorganisms.

[46] Ali U., Saeed M., Ahmad Z., Shah F.-u.-H., Rehman M.A., Mehmood T., Waseem M., Hafeez H., Azam M., Rahman A. (2023). Stability and Survivability of Alginate Gum-Coated *Lactobacillus rhamnosus* GG in Simulated Gastrointestinal Conditions and Probiotic Juice Development. J. Food Qual..

[47] Keleszade E., Kolida S., Costabile A. (2022). The cholesterol lowering efficacy of *Lactobacillus plantarum* ECGC 13110402 in hypercholesterolemic adults: A double-blind, randomized, placebo controlled, pilot human intervention study. J. Funct. Foods.

[48] Singhal N., Maurya A.K., Mohanty S., Kumar M., Virdi J.S. (2019). Evaluation of Bile Salt Hydrolases, Cholesterol-Lowering Capabilities, and Probiotic Potential of *Enterococcus faecium* Isolated From Rhizosphere. Front. Microbiol..

[49] Sengun I.Y., Yalcin H.T., Kilic G., Ozturk B., Peker A.K., Terzi Y., Atlama K. (2024). Identification of lactic acid bacteria found in traditional Shalgam juice using 16S rRNA sequencing and evaluation of their probiotic potential in vitro. Food Biosci..

[50] Bartram E., Asai M., Gabant P., Wigneshweraraj S. (2023). Enhancing the antibacterial function of probiotic Escherichia coli Nissle: When less is more. Appl. Environ. Microbiol..

[51] Papavasileiou K., Papavasileiou E., Tseleni-Kotsovili A., Bersimis S., Nicolaou C., Ioannidis A., Chatzipanagiotou S. (2010). Comparative antimicrobial susceptibility of biofilm versus planktonic forms of *Salmonella enterica* strains isolated from children with gastroenteritis. Eur. J. Clin. Microbiol. Infect. Dis..

[52] Perswani P., Ismail S.M., Mumtaz H., Uddin N., Asfand M., Bin Khalil A.B., Ijlal A., Khan S.E., Usman M., Younas H. (2024). Rethinking HDL-C: An In-Depth Narrative Review of Its Role in Cardiovascular Health. Curr. Probl. Cardiol..

[53] Hakkak R., Gauss C.H., Bell A., Korourian S. (2018). Short-Term Soy Protein Isolate Feeding Prevents Liver Steatosis and Reduces Serum ALT and AST Levels in Obese Female Zucker Rats. Biomedicines.

[54] Herrera M.D., Pérez-Ramírez I.F., Reynoso-Camacho R., Reveles-Torres L.R., Servín-Palestina M., Granados-López A.J., Reyes-Estrada C.A., López J.A. (2023). Chemometric Evaluation of RI-Induced Phytochemicals in Phaseolus vulgaris Seeds Indicate an Improvement on Liver Enzymes in Obese Rats. Molecules.

[55] Schauss A.G., Merkel D.J., Glaza S.M., Sorenson S.R. (2007). Acute and subchronic oral toxicity studies in rats of a hydrolyzed chicken sternal cartilage preparation. Food Chem. Toxicol..

[56] Guo Y., Liu J., Tuo Q., Zhang D., Wanapat M., Xin G. (2024). The effect of dietary supplementation of *Lycium barbarum* leaves on the growth performance, organ indexes and intestinal microflora of rats. Front. Vet. Sci..

