# Supplementary material for: A Comprehensive Safety Assessment of Ralstonia eutropha H16 for Food Applications: Integrating Genomic, Phenotypic, and Toxicological Analyzes
Source: Microorganisms. 2025 Jun 6;13(6):1323. doi: 10.3390/microorganisms13061323 (PMC12195361; doi:10.3390/microorganisms13061323)
Supplement: Supplementary file 1 [file microorganisms-13-01323-s001.zip › microorganisms-3643594-supplementary.pdf]

microorganisms

MDPI

Supplemental Materials

A Comprehensive Safety Assessment of *Ralstonia eutropha* H16 for Food Applications: Integrating Genomic, Phenotypic, and Toxicological Analyzes

Xiaoyan You [1,2,3,*,†], Shuxia Song [1,2,3,†], Bing Li [2], Hui Wang [2,4], Le Zhang [1,2], Xiangyang Li [1,2], Junliang Chen [1], Zhiguang Zhu [2] and Guoping Zhao [2,5,6,7,*]

[1] Henan Engineering Research Center of Food Microbiology, College of Food and Bioengineering, Henan University of Science and Technology, Luoyang 471023, China; asongshuxia@163.com (S.S.); zhangle@tib.cas.cn (L.Z.); xiangyang_224@163.com (X.L.); junliangchen@126.com (J.C.)
[2] National Center of Technology Innovation for Synthetic Biology, Tianjin Institute of Industrial Biotechnology, Chinese Academy of Sciences (CAS), Tianjin 300308, China; libing@tib.cas.cn (B.L.); wanghuih@tju.edu.cn (H.W.); zhu_zg@tib.cas.cn (Z.Z.)
[3] Haihe Laboratory of Synthetic Biology, Tianjin 300308, China
[4] School of Life Sciences, Faculty of Medicine, Tianjin Key Laboratory of Function and Application of Biological Macromolecular Structures, Tianjin University, Tianjin 300072, China
[5] CAS-Key Laboratory of Synthetic Biology, CAS Center for Excellence in Molecular Plant Sciences, Institute of Plant Physiology and Ecology, Chinese Academy of Sciences, Shanghai 200032, China
[6] CAS Key Laboratory of Quantitative Engineering Biology, Shenzhen Institute of Synthetic Biology, Shenzhen Institute of Advanced Technology, Chinese Academy of Sciences, Shenzhen 518055, China
[7] Engineering Laboratory for Nutrition, Shanghai Institute of Nutrition and Health, Chinese Academy of Sciences, Shanghai 200031, China
* Correspondence: xiaoyanyou@haust.edu.cn (X.Y.); gpzhao@sibs.ac.cn (G.Z.)
† These authors contributed equally to this work.

Supplemental Tables:

Supplemental Table S1 Criteria for determination of drug sensitive paper

Antibiotic	Concentration (μg/piece)	Criteria for judging the diameter of the inhibition circle (mm)		
		R	I	S
Kanamycin	30	⩽13	14-17	⩾18
Ampicillin	10	⩽13	14-16	⩾17
Gentamicin	10	⩽12	13-14	⩾15
Streptomycin	10	⩽11	12-14	⩾15
Chloramphenicol	30	⩽12	13-17	⩾18
Tetracycline	30	⩽12	13-18	⩾19
Clindamycin	2	⩽14	15-20	⩾21
Ciprofloxacin	5	⩽15	16-20	⩾21
Erythromycin	15	⩽13	14-22	⩾23
Vancomycin	30	⩽14	15-16	⩾17

“R” indicates resistant; “I” indicates moderately sensitive; “S” indicates sensitive.

Supplemental Table S2 List of virulence factor identified in the *Ralstonia eutropha* H16 genome.

Source	Gene	Production/Function	Classification	Subject Coverage	Query Coverage	Identity	E-Value
VFDB	wbpA	UDP-N-acetyl-D-glucosamine 6-dehydrogenase	Immune modulation	81.86	81.86	81.9	9.83E-267
VFDB	STM0274	putative cytoplasmic protein	Effector delivery system	80.89	81.3	80.6	2.05E-302
PHIB	iscU	Fe-S cluster sensor	Salmonellosis	81.89	81.89	81.9	2.20E-73
PHIB	rplN	50S ribosomal protein	Pertussis	87.7	87.7	87.7	4.91E-73
PHIB	ompA	Outer membrane protein A	Bacterial wilt	84.79	84.33	84.8	7.52E-114
PHIB	paaB	phenylacetic acid catabolism	Cystic fibrosis	84.78	84.78	84.8	2.31E-56
PHIB	paaA	Phenylacetic acid degradation protein	Cystic fibrosis-like disease	82.92	84.16	82.5	1.75E-203
PHIB	lsfA	1-Cys peroxiredoxin with thioldependent peroxidase activity	Opportunistic infection	83.96	83.96	84	5.76E-136
PHIB	BPSL2748	Alkyl hydroperoxide reductase C	Melioidosis	82.55	82.55	82.5	4.73E-135
PHIB	treS	Trehalose synthase	Bacterial wilt	80.89	81.85	80.2	9.02e-316
PHIB	RSc0454	FAD-linked oxidase	Bacterial wilt	87.96	86.39	86.6	0.00E+00
PHIB	gyrA	DNA gyrase	Bacterial grain rot	84.45	85.27	83.6	0.00E+00
PHIB	GyrA	DNA gyrase (bacterial topoisomerase II)	Bacterial grain rot	84.45	85.27	83.6	0.00E+00
PHIB	GyrA	DNA gyrase (bacterial topoisomerase II)	Bacterial grain rot	84.34	85.15	83.5	0.00E+00
PHIB	GyrA	DNA gyrase (bacterial topoisomerase II)	Bacterial grain rot	84.22	85.03	83.4	0.00E+00
PHIB	GyrA	DNA gyrase (bacterial topoisomerase II)	Bacterial grain rot	84.22	85.03	83.4	0.00E+00
PHIB	hfq1 (bglu_1g14550)	RNA-binding protein Hfq	Bacterial panicle blight (rice)	94.59	94.59	94.6	7.00E-45
PHIB	Hfq	RNA-binding protein	Pertussis	89.74	89.74	89.7	2.40E-44
PHIB	Hfq	RNA Chaperone	Pertussis	89.74	89.74	89.7	2.40E-44
PHIB	hfq	RNA chaperone	Pleuropneumonia (pig)	80.6	80.6	80.6	1.46E-34

PHIB	hfq	Regulators of systemic infection	Food poisoning	82.09	82.09	82.1	2.77E-34
PHIB	hfq (PANA_RS17940)	RNA-binding protein	Center rot (onion)	82.09	82.09	82.1	2.93E-34
PHIB	hfq	Multiple regulator	Yersiniosis	80.6	80.6	80.6	3.82E-34
PHIB	Hfq	RNA-binding protein	Pneumonic plague	80.6	80.6	80.6	3.82E-34
PHIB	speC (RSc2365)	Ornithine decarboxylase	Bacterial wilt	88.14	87.48	87.6	0.00E+00
PHIB	STM14_1005	Putative acyl-CoA dehydrogenase	Salmonellosis	81.72	81.72	81.7	1.83E-238
PHIB	aroG1	Phospho-2-dehydro-3-deoxyheptonate aldolase	Bacterial wilt	85.79	85.79	85.8	1.02E-230
MvirDB	NP_287086.1	urease subunit gamma [Escherichia coli O157:H7 EDL933]	pathogenicity island	80	80	80	3.17E-52
MvirDB	NP_286678.1	urease subunit gamma [Escherichia coli O157:H7 EDL933]	pathogenicity island	80	80	80	3.17E-52
MvirDB	NP_309349.1	urease subunit gamma [Escherichia coli O157:H7 str. Sakai]	pathogenicity island	80	80	80	3.17E-52
MvirDB	secG	general secretory pathway, protein-export membrane protein	virulence protein	82.42	82.42	82.4	1.71E-42
MvirDB	Q8XU35	SubName: Full=Probable histone-like dna-binding protein hu-beta (Ns1);	virulence protein	87.91	87.91	87.9	1.72E-50
MvirDB	Q8XU35	SubName: Full=Probable histone-like dna-binding protein hu-beta (Ns1);	virulence protein	87.91	87.91	87.9	1.72E-50
MvirDB	P0ACS9	RecName: Full=HTH-type transcriptional regulator AcrR; AltName: Full=Potential acrAB operon repressor;	transcription factor	83.33	83.33	83.3	1.10E-07
MvirDB	P0ACS9	RecName: Full=HTH-type transcriptional regulator AcrR; AltName: Full=Potential acrAB operon repressor;	transcription factor	83.33	83.33	83.3	1.10E-07
MvirDB	rmlA	glucose-1-phosphate thymidylyltransferase	virulence protein	82.76	82.76	82.8	3.47E-181
MvirDB	sodB	putative superoxide dismutase	virulence protein	83.85	83.85	83.9	1.70E-123
MvirDB	secY	preprotein translocase SecY subunit	virulence protein	81.98	81.53	82	2.98E-248
MvirDB	NP_462660.1	putative inner membrane protein [Salmonella typhimurium LT2]	virulence protein	84.89	84.89	84.9	1.32E-134

MvirDB	NP_462660.1	putative inner membrane protein [Salmonella typhimurium LT2]	virulence protein	84.89	84.89	84.9	1.32E-134
MvirDB	NP_459926.1	SlsA [Salmonella enterica subsp. enterica serovar Typhimurium str. LT2]	pathogenicity island	84.89	84.89	84.9	1.04E-133
MvirDB	NP_455439.1	hypothetical protein STY0948 [Salmonella enterica subsp. enterica serovar Typhi str. CT18]	pathogenicity island	84.44	84.44	84.4	6.03E-133
MvirDB	NP_805747.1	hypothetical protein t1983 [Salmonella enterica subsp. enterica serovar Typhi str. Ty2]	pathogenicity island	84.44	84.44	84.4	6.03E-133
MvirDB	Q8Y0Y3	RecName: Full=Integration host factor subunit beta; Short=IHF-beta;	transcription factor	95.7	95.7	95.7	1.45E-57
MvirDB	Q00514	RecName: Full=General secretion pathway protein G; AltName: Full=PilD-dependent protein pddA; Flags: Precursor;	virulence protein	84.62	84.62	84.6	2.10E-06
MvirDB	Q8XWF3	SubName: Full=DNA-binding protein HU-beta, NS1 (HU-1), plays a role in DNA replication and in rpo translation; SubName: Full=Dna-binding protein hu-beta (Ns1) (Hu-1); SubName: Full=Probable dna-binding protein hu-beta (Ns1) (Hu-1);	virulence protein	82.42	82.42	82.4	1.44E-45
MvirDB	Q8XWF3	SubName: Full=DNA-binding protein HU-beta, NS1 (HU-1), plays a role in DNA replication and in rpo translation; SubName: Full=Dna-binding protein hu-beta (Ns1) (Hu-1); SubName: Full=Probable dna-binding protein hu-beta (Ns1) (Hu-1);	virulence protein	82.42	82.42	82.4	1.44E-45
MvirDB	oxyR	oxidative stress regulatory protein	transcription factor	81.15	79.55	80.2	1.47E-172
MvirDB	P29267	RecName: Full=Hydrogenase transcriptional regulatory protein hoxA;	transcription factor	100	100	100	0.00E+00
MvirDB	P23536	RecName: Full=Phosphoenolpyruvate-protein phosphotransferase; EC=2.7.3.9; AltName:	virulence protein	100	100	100	0.00E+00

		Full=Phosphotransferase system, enzyme I; Short=Protein I;					
MvirDB	C7C420	SubName: Full=Putative efflux protein; Flags: Fragment;	antibiotic resistance	89.23	89.23	89.2	2.79E-32
MvirDB	Q8XVW1	SubName: Full=Probable-export membrane transmembrane protein;	virulence protein	83.49	83.8	83.5	9.03E-188
MvirDB	P44973	RecName: Full=Inner membrane protein oxaA;	virulence protein	83.33	83.33	83.3	6.14E-09
MvirDB	O84957	SubName: Full=Positive phenol-degradative gene regulator;	transcription factor	84.66	84.48	84.7	0.00E+00
MvirDB	CAC03612.1	putative aspartate amino transferase [Kluyvera ascorbata]	antibiotic resistance	81.75	81.75	81.8	1.74E-247
MvirDB	Q9K4U8	RecName: Full=Nitric oxide reductase transcription regulator norR2;	transcription factor	100	100	100	0.00E+00
MvirDB	Q9K4U8	RecName: Full=Nitric oxide reductase transcription regulator norR2;	transcription factor	88.76	87.79	87.6	5.94e-316
MvirDB	Q8XU35	SubName: Full=Probable histone-like dna-binding protein hu-beta (Ns1);	virulence protein	81.32	81.32	81.3	2.35E-46
MvirDB	Q8XU35	SubName: Full=Probable histone-like dna-binding protein hu-beta (Ns1);	virulence protein	81.32	81.32	81.3	2.35E-46
MvirDB	O30337	RecName: Full=Urease subunit alpha; EC=3.5.1.5;	virulence protein	99.65	99.82	99.6	0.00E+00
MvirDB	O30337	RecName: Full=Urease subunit alpha; EC=3.5.1.5;	virulence protein	99.65	99.82	99.6	0.00E+00
MvirDB	Q8XXT1	RecName: Full=Urease subunit alpha; EC=3.5.1.5; AltName: Full=Urea amidohydrolase subunit alpha;	virulence protein	86.71	86.71	86.7	0.00E+00

Supplemental Table S3 List of antibiotic resistance genes identified in the *Ralstonia eutropha* H16 genome.

Source	Gene	Production/Function	Classification	Subject Coverage	Query Coverage	Identity	E-Value
ARDB	Q0K7S4	bacitracin	baca	100	100	100	3.32E-210
ARDB	CAJ93947	bacitracin	baca	100	100	100	5.38E-210
ARDB	YP_002006347	bacitracin	baca	97.95	97.95	98	4.34E-206
ARDB	YP_584847	bacitracin	baca	89.76	89.76	89.8	1.12E-192
ARDB	YP_294981	bacitracin	baca	89.76	89.76	89.8	5.32E-191
Resfams	APH3	Aminoglycoside Modifying Enzyme Phosphotransferase	aminoglycoside phosphotransferase	86.29	86.29	86.3	6.88E-75
Resfams	APH3	Aminoglycoside Modifying Enzyme Phosphotransferase	aminoglycoside phosphotransferase	86.29	86.29	86.3	6.88E-75

Supplemental Table S4 Organ coefficients of acute toxicity.

Parameters	Con-Male	RH16-Male	Con-Female	RH16-Female
Heart	0.38±0.03	0.39±0.03	0.40±0.02	0.43±0.07
Thymus	0.16±0.03	0.17±0.02	0.20±0.05	0.21±0.03
Liver	4.07±0.17	4.39±0.13*	3.77±0.32	3.90±0.11
Spleen	0.19±0.02	0.22±0.04	0.22±0.02	0.21±0.02
Pancreas	0.22±0.04	0.24±0.06	0.25±0.08	0.31±0.03
Kidney	0.71±0.04	0.75±0.07	0.66±0.03	0.75±0.05*
Testis	0.65±0.28	0.94±0.08	—	—
Ovaries	—	—	0.05±0.00	0.05±0.01

Results are expressed as mean ± SD (male/female n = 5). * Significantly different from the control group, $P < 0.05$.

Supplemental Table S5 Hematological result of acute toxicity

Parameters	Con-male	RH16-male	Con-female	RH16-female
WBC (10^9/L)	8.60±2.00	9.72±1.94	10.3±2.79	9.56±1.93
Lymph (10^9/L)	7.06±1.69	8.02±1.68	8.68±2.04	7.66±1.38
Mon (10^9/L)	0.18±0.04	0.18±0.08	0.20±0.12	0.24±0.09
Gran (10^9/L)	1.36±0.34	1.52±0.40	1.42±0.69	1.66±0.58
Lymph% (%)	82.02±2.56	82.30±3.62	84.78±3.51	80.68±3.76
Mon% (%)	1.98±0.20	2.12±0.51	1.92±0.62	2.62±0.49
Gran% (%)	16.00±2.47	15.58±3.34	13.30±2.93	16.70±3.50

RBC (10^{12}/L)	6.89±0.29	6.95±0.28	7.46±0.55	7.05±0.24
HGB (g/L)	138.6±4.04	141.4±6.35	143.6±8.50	137.4±4.77
HCT (%)	44.72±1.21	45.08±1.57	45.26±2.68	43.38±1.10
MCV (fL)	65.00±2.18	65.00±1.25	60.82±1.47	61.66±2.17
MCH (pg)	22.08±4.55	20.32±0.50	19.22±0.35	19.46±0.68
MCHC (g/L)	309.6±2.70	313.4±6.11	317.0±4.18	316.0±4.24
RDW (%)	13.06±1.28	13.54±1.04	10.78±0.13	11.26±0.71
PLT (10^9/L)	1347.7±153.42	1281.4±73.79	1018.2±151.99	1020.4±187.09
MPV (fL)	6.10±0.19	5.72±0.18*	5.78±0.13	5.76±0.30
PDW	16.18±0.22	16.08±0.08	16.22±0.24	16.18±0.19

Results are expressed as mean ± SD (male/female n = 5). * Significantly different from the control group, $P < 0.05$. WBC white blood cell count; Lymph Lymphocyte count; Mon monocyte count; Gran neutrophil count; Lymph% lymphocyte percentage; Mon% monocyte percentage; Gran% neutrophil percentage; RBC red blood cell count; HGB hemoglobin; HCT erythrocyte pressure volume; MCV mean erythrocyte volume; MCH mean erythrocyte hemoglobin content; MCHC mean erythrocyte hemoglobin concentration; RDW coefficient of variation of red cell distribution width; PLT platelet number; MPV mean platelet volume; PDW platelet distribution width

Supplemental Figure:

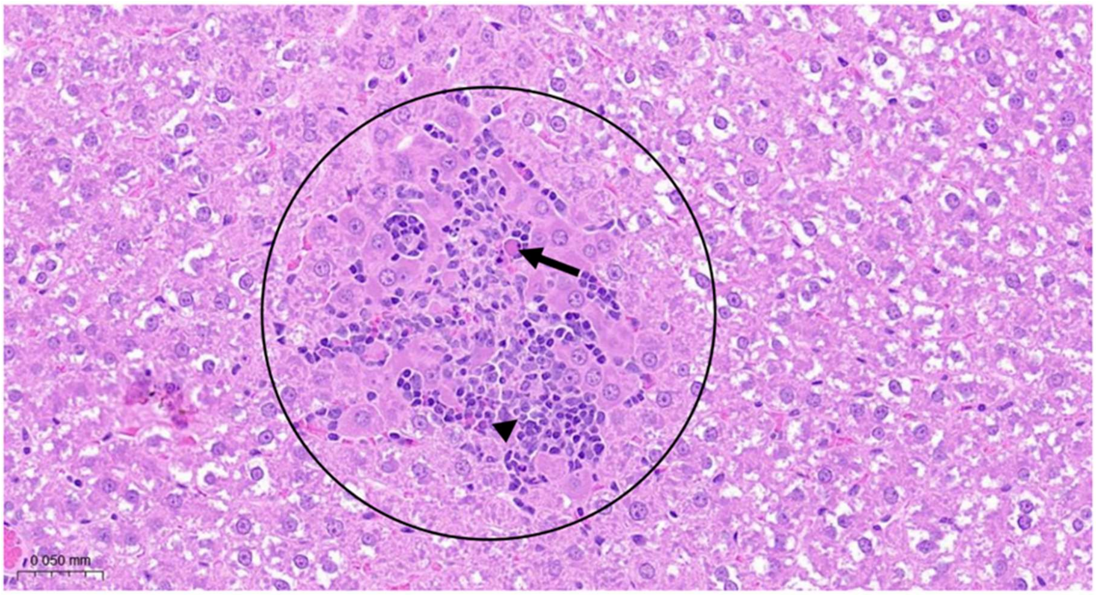

Supplemental Figure S1 Histopathological of liver with acute toxicity control group. Necrotic area of liver cells (black circle), necrotic liver cells (long tail arrow), infiltration of monocytes (no tail arrow), 40x.
